# Regtech: steering the regulatory spaceship in the right direction?

**DOI:** 10.1007/s42786-022-00038-9

**Published:** 2022-02-25

**Authors:** Alexandros A. Papantoniou

**Affiliations:** Papantoniou & Papantoniou LLC, Lamprou Katsoni 8, 1082 Nicosia, Cyprus

**Keywords:** Regtech, Regulation technology, Fintech, Suptech, DLT, Black-box

## Abstract

A spaceship steers by changing its existing orbit or trajectory. The current regulatory framework for financial institutions, albeit essential, is inefficient, thus steering the regulatory spaceship out of orbit. Regtech is an ever-evolving innovation which may have the potential to become a catalyst in financial services industry regulation. It provides automated solutions for monitoring, reporting and analysing regulatory requirements, thereby significantly reducing costs and improving productivity. This paper, however, argues that Regtech currently lacks the envisaged effective and widespread adoption because of risks, ambiguities, costs and difficulties associated with its application. The future of Regtech, therefore, remains uncertain. Yet through careful modification and implementation of recommendation initiatives, Regtech will be able to transform the way in which regulators and financial institutions observe and implement the current regulatory landscape, steering the regulatory spaceship in the right direction.

“I have a dream. It is futuristic, but realistic. It involves a Star Trek chair and a bank of monitors. It would involve tracking the global flow of funds in close to real time, in much the same way as happens with global weather systems and global internet traffic. Its centre piece would be a global map of financial flows, charting spill-overs and correlations” [1].

## Introduction

The financial services industry has witnessed first-hand the revolution of new disruptive technologies. Apart from Blockchain and Fintech, however, the hype of most disruptive technologies^1^ was short-lived. In a world where most buzzwords fizzle out, the inherent scepticism towards new technologies is entirely logical. Yet Regtech proved that it is here to stay. Innovations in Artificial Intelligence (‘AI’) and Distributed Ledger Technology (‘DLT’) underpinned by cloud computing have paved the way for digital regulatory compliance and reporting [2]. This paper examines whether this technology is able to transform the ways in which financial incumbents comply with regulatory requirements and in turn, the supervision methods by regulators.

Regtech has been developed as a response to the inefficiencies generated by the current regulatory framework. Section 1 analyses the factors which render the current regulatory framework both inefficient and unsatisfactory. Manual compliance and regulatory reporting require significant time, contribute towards wasted costs and are more susceptible to human errors. Equally, siloed reporting systems and reporting frameworks have the propensity to produce duplicative data which burdens both financial institutions and regulators if that data are not easily accessible in the financial institution’s systems. This provided the necessary incentives for financial incumbents to seek alternative means of compliance.

Section 2 explores the Regtech phenomenon. It acknowledges that the technology existed prior to the 2008 Global Financial Crisis (‘GFC’) but the aftermath of the crisis was the primary impetus for change. The Post-GFC regulatory influx resulted in significant compliance and supervision costs. Regtech offers a coping mechanism for the ever-evolving regulatory framework by providing enhanced analytics, real-time monitoring and automated reporting. Section 2 suggests that there are two different perceptions to the potential of this technology. Regtech pragmatists view the technology as a mere efficiency facilitator to compliance. In contrast, the technology has also received an evangelical following by Regtech idealists who perceive Regtech as a catalyst bound to enable transformation of financial services industry compliance.

Section 3 examines the potential of Regtech. Primarily, the technology facilitates compliance with regulatory requirements. This technology has enabled several financial institutions to comply with international standards and domestic regulations issued at a national level to reduce costs and time whilst maximising productive allocation of employees. Regtech has also been deployed as a response to the scourge of financial crime. By automatically identifying suspicious transactions, Regtech ensures that fewer criminals remain undetected. Equally, by providing a single interface for identifying and monitoring cross-border transactions, it aims to address complications in cross-jurisdiction compliance. Know-Your-Customer (‘KYC’) and Customer Due Diligence (‘CDD’) processes are also simplified by Regtech solutions as it provides the tools to keep in pace with the digitalised transformation of the financial services industry. This paper also analyses Regtech as a response to the risks posed by Fintech. It is argued that the technology offers the tools to financial incumbents and regulators to reduce exploitation by disruptive technologies and address the threats to financial stability. Lastly, this paper explores the recent innovation of regulatory sandboxes. It is argued that sandboxes enable Regtech specialists to test their solutions in a controlled environment and at the same time provide a platform to regulators to test new and flexible methods of supervision and big data analysis. Overall, Sect. 3 hints that Regtech may indeed have the potential to transform the financial services industry.

Section 4 performs a reality check on this technology and demonstrates that Regtech’s catalytic potential is currently limited. Potential errors in Regtech software code inevitably pose significant operational and systemic risks for the financial services industry. These risks are exacerbated when one realises that market participants do not devote sufficient funds and time for Regtech investment, staff training and in-house development. Even if some market actors significantly increase investment, the fact that Regtech does not guarantee fully automated regulatory compliance may dissuade several financial incumbents from adopting this technology. A more complicated limitation to Regtech’s catalytic potential is proper implementation and integration of the technology. Regtech requires proper implementation in financial institutions systems, otherwise it risks generating compliance breaches with other regulations. Crucially, integration in already established financial institutions would be an immeasurable challenge as it would require an outright replacement of existing systems which so far have been reliable. On a technical level, the limitations of AI also raise issues of integrating the law with technology. Moreover, the current fragmentation of the cross-jurisdictional regulatory framework suggests that Regtech solutions cannot promise consistent compliance for financial service providers operating in multiple markets. Lastly, Regtech software may be hardwired with automated biases thereby circumventing legal and constitutional principles. Thus, it may develop a ‘black-box’ approach by taking decisions which are incomprehensible to computer programmers and regulators. As a corollary, these factors restrict Regtech from achieving its full potential.

Finally, Sect. 5 explores what the future holds for Regtech. To unlock Regtech’s catalytic potential, it is necessary for market participants to lobby for greater harmonisation of financial markets and orchestrate an international common approach. This will improve compliance, information sharing, and consistent regulatory reporting underpinned by Regtech solutions. Equally, closing the gaps in international financial crime regulations will allow Regtech to manage risks to market integrity through a common international approach. This could be facilitated by a new specialised European Anti-Money Laundering (‘AML’) body addressing regulatory fragmentation and educating market actors on efficient Regtech development, integration and operation. Ultimately, standardisation of legal terminology and classification of actors would also help to unlock Regtech’s potential. It would facilitate communication among financial incumbents and regulators thereby resulting in consistent digital regulatory compliance. Lastly, Sect. 5 unravels the concept of ‘Embedded Supervision’ and predicts that it will be critical in influencing Regtech’s future development. This paper concludes that although the future is uncertain, Regtech can steer the regulatory spaceship in the right direction through the adoption of these recommendations.

## The inefficiency of the current regulatory framework

Why do we need financial regulation? What societal goals does it seek to achieve? Financial regulation exists solely to underpin and overall improve the functioning of the financial system [3]. Economic theorists and practitioners have developed different views on the categorisation of the societal goals of financial regulation. A holistic categorisation of the main sources of financial regulation is: (i) protection of investors and other users of the financial system; (ii) consumer protection; (iii) financial stability; (iv) market efficiency; (v) competition and the (vi) prevention of financial crime [3]. In practice, these goals are achieved through specific mandatory legal and reporting requirements imposed on financial institutions. Financial supervision is conducted domestically underpinned by international standards guiding national regulations.

Even though compliance with the regulatory framework is vital to safeguard the functioning of the financial system, it nevertheless requires massive administrative tasks by financial incumbents. Firstly, regulations have to be manually imported into firms’ systems and controls. For example, employees in several financial institutions have to review financial regulations in order to determine “which ones are relevant to their business, and to match the language used within these documents with the terminology (or taxonomy) used within their organisation” [4]. This is time consuming and susceptible to errors as it is only natural for employees to err when importing and transplanting regulations word-for-word into business systems [2]. This issue is aggravated by the international nature of several regulations. Given that international standards and legal reporting requirements vary in cross-border transactions, manual reporting is bound to result in inconsistencies and errors in compliance within a given regulatory framework.

Manual compliance with regulations inherently generates duplicative data in businesses systems [2]. For example, an employee could have reported data in an effort to comply with Market Abuse Regulation 2016 (‘MAR’) but another employee could have already reported some of that data for Markets in Financial Instruments Directive 2004 (‘MiFID II’) purposes. Duplicative data represents an operational nuisance to businesses since it contributes to wasted costs and produces allocative inefficiencies within a firm’s business model. Similarly, manual reporting may result in the transmission of erroneous or inconsistent data to regulators causing delays to data processing and analysis [2]. In turn, this would inevitably require data cleansing or even re-submission which disadvantages both regulators and firms [2].

Regulatory compliance solutions firms aimed to capitalise on the regulatory gap created by these deficiencies through innovative technologies. Indeed, Regtech providers have penetrated this niche market by providing a technology-enabled solution to regulation compliance, the value of which is widely appreciated by both regulators and financial institutions [5]. Prior to examining the particulars of Regtech, it is important to understand the origins and operational framework of this technology.

## The regtech phenomenon

### Origins

Regtech is a contraction of the terms ‘regulatory and technology’ meaning that it involves reliance on technology for regulatory and compliance purposes [6]. Regtech therefore, represents a technology-enabled solution for the management of regulatory procedures within the financial industry. The emergence of this technology can be attributed to several factors. Even if the roots of Regtech can be traced before 2008, the massive regulatory burdens incurred by financial institutions and regulators caused by the GFC aftermath have been the primary impetus behind the Regtech industry [7]. The GFC contributed towards a “complex, fragmented and ever-evolving” regulatory environment [8]. It gave rise to a regulatory influx whereby complex and prescriptive regulations needed to be implemented by financial institutions and overseen by regulators, leading to a substantial increase in compliance and supervision costs [8]. For example, in 2015 it was reported that global expenditure on governance, risk and compliance amounted to $80 billion and expected to reach $120 billion by the end of 2020 [9]. Consequently, it became clear that the regulatory landscape required an efficient solution to reduce costs by simplifying the compliance and supervision processes.

For financial innovation specialists, the emergence of Regtech was a “predictable response to the post-crisis regulatory agenda” [10]. Indeed, Regtech is an example of ‘regulatory dialectic’ whereby the private sector seeks to offset the consequences of stringent regulatory action imposed by the public sector [10]. Regtech presented the solution to regulatory complexity by providing an accurate, efficient and frequent method of data reporting and analysis [11]. More specifically, Regtech delivers enhanced analytics, real-time information and timely reporting possibilities thereby enabling regulators to provide an adequate response to emerging financial risks. This is extremely important in financial markets where proactive action through on-going monitoring identifies and tackles problems in advance as opposed to retrospective enforcement when the damage has already been done [6]. As the reigns of reporting and analysis shift from human supervision to machines, Regtech represents an entirely novel paradigm which marks a more efficient and effective way of enhancing compliance and monitoring [12].

Before dissecting Regtech’s operational framework, it is of primary importance to distinguish Regtech from Supervisory Technology (‘Suptech’). Whilst Regtech utilises innovative technologies to assist compliance and regulatory reporting by financial institutions, Suptech equips supervisory agencies with innovative technologies to support reporting oversight and regulatory digitisation [13]. Suptech can be deployed by regulators in an effort to enhance efficiency in “reporting and proactively monitoring the risk and compliance of financial institutions” [13]. Suptech enhances “the value of data collected by enriching its intelligibility and interoperability” through simplified visualisation of dense and complicated datasets as well as trend and forecasting analysis [14]. Consequently, Suptech is to be regarded as a close equivalent to Regtech for improving efficiency in monitoring and oversight by supervisory agencies.

### Operational framework

Whilst it is true that financial incumbents have always pursued automated reporting and compliance tools, the GFC aftermath necessitated the automation of regulatory obligation compliance [6]. Consequently, these financial incumbents represent both the primary driver and the central market for Regtech solutions [6]. For a holistic understanding of the reasons why financial institutions have shifted their focus towards technology-enabled regulatory solutions, it is first prudent to discern how the market perceives Regtech’s operational framework.

The operation of Regtech may be perceived through a realistic or an idealistic lens. The realistic perception of Regtech’s operational framework is best articulated by Alam et al. They suggest that financial institutions rely on Regtech software to comply with regulations more efficiently. More importantly, Regtech specialists work together with these institutions and regulators using cloud computing technology and big data to exchange real-time information [15]. In turn, Regtech firms gather information from financial institutions in relation to previous regulatory failures and expose potential compliance risks that the institutions are expected to address [15]. This may encourage a three-way communication between financial institutions, regulators and Regtech firms with a common aim of promoting efficient regulatory monitoring, reporting and data sharing. In this sense, Regtech may be understood as a subset of Fintech which provides a technological solution to efficient regulatory compliance.

Although the realistic perception is certainly accurate, idealists would argue that it nevertheless constitutes a banal way of understanding Regtech. The idealistic view deems the realistic model as rather narrow on the basis that it portrays Regtech as a subset of Fintech, merely facilitating the efficiency of regulatory compliance [16]. In contrast, idealists believe that “Regtech is on the cusp of a breakthrough that has the potential to transform the industry’s approach to compliance and create additional value” [17]. KPMG reported that this breakthrough is evidenced by wide regulatory support, increased investment and demand for data-driven regulatory compliance solutions [17]. Strongly believing in this technological breakthrough, the idealistic view regards Regtech as the “next logical evolution of financial services regulation” which lies at the heart of the financial industry [6]. Idealists regard Regtech as the technological tool which will reform the way in which data are gathered, processed, visualised and understood. Equally, it is believed that the traditional compliance mechanism within financial institutions which is heavily reliant upon human intervention is bound to be disrupted and replaced by automation. Therefore, whilst the realistic view regards Regtech as a mere efficiency facilitator, the idealistic view focuses on Regtech’s catalytic potential to transform the financial services industry. In order to assess this potential, this paper proceeds to conduct an examination of the efficiencies generated by this technology.

## Regtech efficiencies

### Regulatory reporting, compliance and monitoring

The costs associated with regulatory compliance have been described as a “source of disruption” by 87% of banking CEOs [18]. Conversely, the costs associated with non-compliance are also immense. For instance, studies revealed that banks have been charged $200 billion for non-compliance [19]. This threat of non-compliance charges drove several financial institutions such as Citi Bank to employ thousands of staff with the sole responsibility of managing compliance [19]. However, financial institutions are increasingly becoming more cognisant of the fact that manual compliance does not ensure consistent adherence to regulatory obligations which have been the subject of a “ceaseless bombardment of changes” [19]. Consequently, financial incumbents employed Regtech solutions which provide an efficient mechanism to address these costs. In the case of Citi Bank, the costs associated with employing 30,000 regulatory compliance staff certainly outweighed the costs of deploying a Regtech solution firm and implementing their software [19]. Regtech’s potential may therefore be evidenced by the ability to significantly reduce costs, enhance regulatory efficiency and contribute towards better compliance outcomes [20].

More generally, prudential regulation expects financial institutions to “understand, monitor and report all aspects of their activities to regulators in the jurisdictions in which they operate” [21]. Regtech solutions ensure compliance whilst reducing the risk of regulatory breaches, fines and reputational damage by working more accurately and efficiently. For example, a Santander deployed ABACUS/Transactions Regtech solution to comply with ECB’s Money Market Statistical Reporting (‘MMSR’). MMSR compliance is particularly onerous for financial institutions given that it requires reporting of all euro dominated money market transactions to the ECB [22]. Santander would have been forced to expand their compliance department leading to an inefficient allocation of employee resources. Rather, Santander deployed the Regtech solution “with lightning speed, which ensured the daily, automatic and stable delivery of MMSR obligations” [23]. This demonstrates the ability of Regtech to significantly improve the efficiency of financial institutions and the potential to transform regulatory compliance in the financial services industry.

Regtech solutions have also been deployed by financial institutions to achieve compliance with the General Data Protection Regulation (‘GDPR’). GDPR is regarded as one of the strictest privacy and security laws globally by mandating a stringent standard for personal data protection [24]. The introduction of GDPR has been troublesome for the banking industry who was already subject to cumbersome regulations “coming in waves” from different jurisdictions [25]. As Baker accurately submitted, if GDPR compliance was left unresolved, banks would have suffered significant loss in profits and reputational damage [25]. ElinarAI Regtech solution employs AI to mine vast amounts of personal data and identifies potential GDPR data [26]. This solution is particularly advantageous for the banking industry which stores large amounts of customer personal data and would otherwise require days of manual checking, reporting and ongoing monitoring. Therefore, this contributes to the overall argument that Regtech enhances compliance efficiency and also significantly reduces costs.

At the same time, Regtech is also capable of monitoring the performance of financial institutions to ensure financial health. The financial viability of a financial institution is determined through an assessment of solvency, profitability and liquidity ratios. Risk weighted assets, returns on investments and efficiency ratios are also taken into consideration in financial health monitoring. More specifically, Basel III imposes stringent liquidity requirements on financial institutions which entail extensive calculations of their Liquidity Cover Ratios and Net Stable Funding Ratios on an ongoing basis [27]. Regtech solutions providing visual analytics “have the capability to perform trend analysis, sensitivity analysis, scenario analysis, anomaly detection, early warning and predictive modelling across various durations” [28]. For example, the Moody’s Analytics Regtech solution “calculates liquidity ratio coverage, net stable funding ratio and other key metrics within a single platform to help banks optimize their liquidity compliance structures” [29]. This therefore evidences the ability of Regtech solutions to provide the necessary tools for financial health reporting and monitoring.

## AML and KYC compliance

It is common knowledge that money laundering has been a cause for concern in recent years as a result of the expansion of the banking industry which constitutes the involuntary medium for the commitment of these crimes. Singapore, for instance, has been increasingly susceptible to money laundering practices due to its transformation to a leading financial hub of Private and Open Banking systems [30]. Such prominent financial hubs could immensely benefit from deploying Regtech solutions to combat financial crime. This argument is supported by the evident success of several banks who have deployed Regtech software, along with their internal data reporting systems, to address and tackle AML concerns. For example, a bank adopted a cloud-based solution “to review 400,000 SMEs customers, gain a holistic view of risk and establish a robust audit trail” [17]. This reduced the time required to undertake the necessary authorisations, ensuring efficient monitoring of AML practices and providing an insight into customer activities [17].

AML and KYC practices have presented a “fertile area for Regtech development” [8]. AML compliance requires financial incumbents to produce suspicious transactions reports and experts to validate client transactions against several criteria [31]. AML is an extremely demanding sector which requires extensive knowledge and training, which results in experts being limited in numbers and costly to employ [10]. Regtech software completely transforms this process to automated transaction monitoring where suspicious transactions are flagged automatically [31]. This form of Regtech automates the flagging of suspicious transactions using rules-based criteria. Equally, another form of Regtech that may be deployed in this area is one that uses AI to flag such transactions. In this form, the detection of suspicious transactions ceases to “exclusively depend on the attentiveness of individual staff members” and rather, employees are merely in charge of determining whether the flagged transactions are in fact worthy of reporting to regulators [31]. This is a valuable alternative since manual detection of suspicious transaction is a labour-intensive and time-consuming task. Indeed, this argument is even more pertinent in recent years where suspicious transaction reports have increased dramatically^2^ [32]. Through utilising the merits of AI and machine learning, financial institutions are able to adjust their focus solely on precarious transactions which concern serious financial crimes whilst also reducing costs [32].

The potential of Regtech to address AML compliance can be further illustrated by the Westpac Scandal. Westpac Banking Corporation was accused by Australia’s Financial Intelligence Agency of breaching AML finance laws on twenty-three million counts [33]. Westpac’s internal investigation discovered that the breaches were the result of under-developed technology, poor employee attentiveness and a lack of expertise and resources [34]. In the absence of a Regtech solution to flag suspicious transactions, Westpac’s employees’ efforts to identify and tackle regulatory breaches proved to be fruitless. In September 2020, Westpac negotiated to pay a fine of A$1.3bn in what has been described as Australia’s “biggest breach of money laundering laws” [35]. The Westpac scandal implies that reliance on Regtech solutions would also reduce liability risk. This is because automated compliance reduces the risk of human error inherently embedded in manual processes [36]. The law is therefore observed and complied with in a more consistent and systematic manner [31]. Overall, this demonstrates the potential of Regtech to mitigate the risks associated with AML breaches thereby enhancing compliance in the financial services industry.

On an international level, Regtech is equipped to address ambiguities on international AML regulations which result in “fragmentation among jurisdictions and conflicting sets of requirements” [11]. The simplification and instantaneous nature of cross-border transactions inevitably heightens the risk of regulatory breaches. In response, financial institutions have to comply with an array of regulations mandated by different jurisdictions resulting in excessive costs. Regtech aims to provide a “single interface for monitoring cross-border business activities and implementing compliance frameworks” and deliver “automated transaction-based cross-border monitoring” [30]. By offering the ability to reduce cumbersome oversight of cross-jurisdiction regulation compliance, Regtech contributes towards a more stable operation of financial service providers.

In relation to KYC and CDD requirements, one must concede that although they are extremely burdensome and time-consuming, they constitute a prerequisite for access to the banking system. Regtech software is able to address these burdens by “providing aggregated data repositories combined with machine learning to vet entities and individuals against global sanction and watchlists, politically exposed persons and entities, and adverse media and enforcement reports, with real time monitoring and alerts when situations change” [37]. This may prove to be a vital asset as the global financial system begins to leverage Know-Your-Data approach along with the present Know-Your-Customer principles bringing in a more wholesome evaluation [10]. Essentially, this suggests that as the financial services industry “becomes increasingly digitalised and data-driven” [8] there is a need for a solution to understand, analyse and monitor these data. The solution is presented by Regtech’s “increasingly datacentric nature” [8] which provides the tools to accompany and expedite the digitisation of financial service providers.

### Addressing fintech concerns

Regtech has been regarded by several practitioners and academics as the antidote to the risks and challenges generated by the emergence of Fintech. This technology dominates every aspect of the global financial system. In fact, several technology giants such as Alibaba have completely disrupted traditional finance thus, requiring regulators to catch up [6]. Fintech impacts the whole value chain by constantly inventing new methods of payment, credit provision and capital market investment [48]. At the same time, however, the digitisation of the financial industry inevitably means that the financial system becomes more susceptible to theft, fraud and other cybercriminal activities [6]. A pertinent example of this trend is the unregulated space of virtual assets. FATF’s president acknowledged the efficiencies granted to the financial industry through innovative technologies such as blockchain but sounded the alarm on the infinite exploitation possibilities of virtual assets by criminals and terrorists to evade sanctions [39]. This indicates that there is a need for a technology-enabled regulatory solution to oversee and contain the risks caused by Fintech’s exponential growth.

Fintech has arguably disrupted the societal goals that financial regulation seeks to achieve such as financial stability, reduction in financial crime and consumer and investor protection. In detail, fintech has the propensity to undermine financial stability by amplifying financial and operational risks [40]. The rapid growth of the Fintech industry means that individual businesses may not have the necessary risk management expertise and thus, may under-estimate the level of risk they are taking on [40]. For instance, cyber-attacks have been cumulating in recent years and pose a significant threat to the financial system and “fintech could serve to accentuate this risk” [40]. Given that fintech connects and links more institutions together, this increases their susceptibility to cyber-attacks through increased range and number of entry points [40]. Equally, fintech may amplify shocks to the financial system thus raising the likelihood of financial instability. It could be argued that the rapid growth of fintech may also generate greater reputational contagion. If “significant and unexpected losses” are incurred on a single Fintech platform this could be interpreted as indicating potential losses across the sector [40]. Consequently, one could foresee that “increased access, combined with risks like cyber risk that suffer from weak link problems, may also increase contagion risk” [40]. The integration of activities between the Fintech sector and other financial intermediaries has to go stage by stage with the degree of robustness of systems involved. The regulators approach to Regtech could also be at different levels to different sectors. As the financial technology sector relies further on automated solutions and AI,^3^ new risks may be created by the lack of human supervision leading to greater contagion in financial markets [41].

Fintech increases the burden on financial service providers by exposing defects in their current business models and places greater responsibility for effective monitoring on regulators [42]. This is because Fintech poses a “threat to financial stability” because exploitation results in “credit risk, systemic risk and information security risk, as well as providing new opportunities for regulatory arbitrage” [43]. Accordingly, the rapid evolution and development of Fintech required the equal and opposite development of Regtech [6]. One may also go as far as to suggest that “Fintech growth has *elicited* the need for Regtech” [44]. By having in place automated monitoring and regulatory compliance software, Regtech ensures that the business models of financial service providers are sufficiently robust and at the same time, regulators are able to successfully oversee and condemn any potential exploitations by Fintech.

### Regulatory sandboxes

Fintech has proven to be a major challenge both for regulators and the financial services industry. It has been evolving at an unprecedented pace meaning that regulators must rely on technology to effectively monitor compliance with regulatory requirements across such dynamic cross-border markets [6]. However, unlike financial institutions, regulators find themselves in deadlocks. These deadlocks are manifested by the need to implement stringent regulatory oversight to maintain financial stability and consumer protection but also the need to preserve incentivises to innovate in the Fintech sector [45].

One solution to this issue was provided by regulatory sandboxes. The first regulatory sandbox was introduced by the UK Financial Conduct Authority (‘FCA’) in 2015 and has since received wide support by several jurisdictions. In essence, regulatory sandboxes grant the ability to firms, including Regtech specialists, to test innovative products and services in a controlled environment with real consumers [46]. While Regtech development itself is not a challenging task, a critical limitation is the constraint on the regulators’ ability to understand and analyse the increased amount of data generated by these solutions [8]. To address this limitation, the FCA restricts access to its regulatory sandbox for limited businesses with a detailed testing plan [8]. Although this implies that sandboxes may be underdeveloped, it is believed that “the greatest potential for the sandbox tool is in the context of the evolution of Regtech, through the opportunity they present for the testing of new approaches by industry and regulators” [8]. Regulatory sandboxes may significantly reduce asymmetric information as “regulatory oversight and continuous dialogue with the regulator during the testing period offers reassurance to investors that firms meet their regulatory obligations” [47]. As a consequence, successful sandbox businesses receive FCA’s ironclad endorsement that they comply with regulatory and consumer protection requirements and obtain better access to finance since the innovation has been successfully tested. Indeed, start-ups involved in the first cohort of the sandbox received more than £130 million of equity funding [48].

This has been taken a step further by the Global Financial Innovation Network (‘GFIN’) which provides a network of international financial regulators and related organisations to discuss common policy and build a global regulatory sandbox. GFIN aims at expanding product and service testing beyond the country of origin which is highly desirable as most start-ups require global market demand to preserve viability. From a regulator’s perspective, sandboxes are also relied upon for the development of new, innovative and flexible methods [18]. This means that a potential Suptech solution can be tested in the sandbox to ensure that it is sufficiently robust whilst also enhancing data monitoring and analysis. Given the limited capacity of the FCA’s sandbox, a global regulatory sandbox will generate greater possibilities for testing Regtech and Suptech solutions. More specialists will be able to test and obtain approval for their solutions thereby contributing to more innovative compliance mechanisms for financial institutions along with improved supervision and surveillance by regulators.

## Reality check: factors hampering regtech’s potential

Section 3 exemplified that Regtech provides tremendous benefits both for financial institutions and regulators. The idealistic perception of Regtech is based on the premise that these efficiencies are so significant so as to establish this technology as a catalyst in transforming the financial services industry. Nevertheless, the practical reality is that there are several challenges and risks which effectively thwart the realisation of these benefits [49]. Prima facie, there are three objections to the argument for Regtech’s catalytic potential. As Arner et al. accurately submitted: (i) there is a time lag between finding a particular Regtech solution and for the market itself to settle prior to regulatory intervention; (ii) the availability of technology does not guarantee widespread adoption and (iii) there are significant benefits associated with limited regulatory intervention on innovation and technological standards [50]. Section 4 however, proposes an alternative categorisation of the factors hampering Regtech’s potential, namely: (i) Operation and Systemic Risks; (ii) Budget and Skilled Resources; (iii) Automation; (iv) Proper Implementation and Integration; (v) Lack of International Regulatory Harmonisation and Domestic Implementation and (vi) the Possibility of a ‘Black-Box’ Approach.

### Operational and systemic risks

The major weaknesses exposed in Regtech’s operational framework can be boiled down to the fact that the software itself is not subject to regulatory requirements since Regtech solutions do not require authorisation or licence [9]. Consequently, one may reasonably argue that unregulated Regtech specialists providing solutions to financial institutions may prove to be a major cause for concern given that any potential failure means that clients no longer comply with regulatory requirements [9]. The thrust of this argument lies in an analysis of the risks associated with potential errors in Regtech code.

It has been submitted in Sect. 3.2 that Regtech reduces the possibility of human error and thereby liability through automating regulatory compliance. Yet the software only generates these efficiencies if the code itself is accurate [31]. An error in Regtech code creates a greater operational risk than an individual human error because that error will be recurring so long as the software is active [31]. Indeed, as interconnectivity between financial incumbents and regulators increases through regulatory reporting, security risks also increase [10]. More specifically, even a small malfunction in the software’s code may have a “disproportionately large effect, particularly if the firm’s own assurance processes fail to uncover the error for an unreasonable length of time” [49]. This is a major cause for concern given that firms will not be complying with regulatory requirements for that period of time and will therefore be left exposed to financial criminals. Equally important is the fact that errors in Regtech code may also contribute towards an increase in systemic risks. This scenario requires the uniform adoption of a Regtech solution across a particular market. This could be the case where a Regtech specialist achieves market dominance through innovation or where a particular solution has received regulatory endorsement [31]. If the same Regtech solution is widely used throughout a particular market, this means that the adverse consequences of a code error will be shared by all market participants resulting in a systemic compliance collapse [31].

A further complication stemming from errors in Regtech code is the issue of liability. If the code error results in data loss or transaction delay who should be liable to clients for the damage? Should it be the Regtech specialist or the financial institution? It is evident that responsibility and liability of complying with regulations lies solely on financial institutions. If a financial institution relies on technology to enhance compliance, the responsibility and liability of compliance does not shift to the technology company. Yet although the answer seems obvious, lack of enforcement by regulators has led many to argue that the locus of responsibility remains unclear [51]. The UK Treasury Committee acknowledged that there is a lack of enforcement against individuals and institutions for software failings and authoritatively stated that if these failings continue, it will have to question whether regulator powers are sufficiently vigorous for enforcement [52]. This is a reasonable position because it is vital to hold both individuals and financial institutions to account for any potential software failures. This will eliminate the possibility of repeating the same mistakes and shift the attention of management towards “risk of incidents and incident management” [52]. Until and unless regulators hold Regtech specialists and financial institutions accountable for Regtech failings, regulatory mechanisms cannot be said to “have teeth, and equally as importantly, [be] seen to have teeth” [38]. Consequently, this limits Regtech’s catalytic potential in transforming the financial services industry.

### Budget and skilled resources

The director of innovation of the UK FCA conceded that Regtech solutions are not readily available to meet the needs of regulators and financial service providers [46]. Therefore, he urged for Regtech solutions to be advanced in-house [46]. Indeed, given the threat of operational and systemic risks, it is natural for regulators to expect firms to acquire sufficient expertise to be able to ‘second-guess’ Regtech specialists in order to avert a systemic compliance collapse [49]. This requires skilled individuals to build and integrate the appropriate Regtech solutions to be able to understand and simplify the vast amounts of data regulators have to grapple with [46]. However, it also requires sufficient investment and resources for individuals to obtain the necessary skills to minimise potential risks associated with code errors.

It is certainly vital to enhance and widen the board’s skill set on Regtech solutions to ensure that well-informed decisions are being made, technology risks are appropriately assessed, and senior managers are sufficiently skilled to discharge their responsibilities [49]. Firms, regulators, risk and compliance practitioners, consultancies and systemically important financial institutions have acknowledged that underinvestment and lack of training impeded widespread Regtech adoption. A survey conducted by Thomson Reuters discovered that 34% of firms believed that lack of investment was thwarting the process of deploying Regtech solutions [49]. Furthermore, 27% identified lack of in-house skills as a factor for not utilising Regtech [49]. This demonstrates that Regtech cannot be the catalyst in transforming the financial services industry unless these institutions significantly invest in Regtech solutions and in-house training. The percentage of systemically important financial institutions who have invested or appointed Regtech specialists at board level has nevertheless grown from 2% in 2018 to 21% in 2019 [49]. These institutions have at the same time widened the skill set at board level from 32% in 2018 to 52% in 2019 [49]. This exemplifies that financial institutions recognise the importance of Regtech and one should expect this trend to maintain an upward trajectory in the following years as more institutions increase investment to enjoy the efficiencies of this technology. Yet it is equally important to note that although there is a trend in increased investment and training for Regtech solutions, a lot more is needed to conclude that Regtech is able to transform the financial services industry.

### Automation

Automation^4^ is an extremely sought-after feature in the financial services industry since it contributes to lower costs and improved efficiency. Logic dictates that Regtech ought to be equally sought-after by providing the ability to monitor and report regulatory requirements automatically. However, that is only part of the story. Although Regtech promises automation, it is debatable whether regulatory and compliance systems can be fully automated [54]. For example, when identifying and flagging suspicious transactions for AML, Regtech inevitably requires human input to oversee and produce a report on these transactions. The current limitations of AI and machine learning indicate that Regtech cannot be fully automated as it is capable of merely identifying suspicious transactions without comprehending the reasoning behind its actions.

This argument does not negate that Regtech represents a ‘state-of-the-art’ technology. It does, however, hint that it will require greater efforts to change the mindsets and perceptions of regulators and financial institutions to use Regtech. One cannot doubt that “it takes time and effort to convince people that a new technology is worth the cost, the effort or the potential risk” [55]. In relation to Regtech, these efforts will have to be significantly greater given that monitoring, reporting and data analysis is not in reality fully automated and still requires manual checking. While competition could be one aspect driving adoption of Regtech and AI, evangelising its benefits through learning sessions can reduce the resistance to change. At the same time, failure by market actors to adopt this new digitalised infrastructure could exacerbate “business risk that may separate winners from losers in the coming years” [10]. In an effort to stay ahead of the game, financial institutions may therefore be incentivised to adopt Regtech. Yet even if the necessary incentives are obtained, this leads to issues of proper implementation and integration of this technology.

### Proper implementation and integration

A pressing question for successful Regtech adoption is “how do you verify the machines—not only that the code accurately models regulatory requirements but whether the systems are properly implemented inside the financial institution?" [56]. Integrating the Regtech systems with the financial institutions’ internal systems, with stringent validation process, will give more accurate data. The implementation of new technologies is a “long-term process” and “ongoing refinements” [9] are necessary to ensure that IT systems are accurately integrated. Indeed, it is prudent to question how Regtech systems can be properly integrated in circumstances where they have to fulfil two competing regulatory requirements. Labbé accurately exemplified that whilst MiFID II requires disclosure of an investor’s personal details to the regulator, a failure to notify the investor of that disclosure results in a GDPR breach [9]. Accordingly, the overlap between regulatory disclosures means that companies may find themselves in breach of one regulation in an effort to comply with another [9]. This demonstrates that successful Regtech implementation requires a holistic view of regulatory regimes and proper integration within institutions IT systems.

These concerns are exacerbated if one pays heed to the dichotomy between start-up financial service providers and older established providers. Start-up financial service providers such as Monzo and Revolut will certainly find it easier to integrate Regtech in their day-to-day business activities. This is because their entire business model and operation can simply be compatible with Regtech from the beginning. In contrast, large financial institutions such as Barclays are “somehow chained to their old hardware” [57]. Such institutions would face an immeasurable challenge to integrate Regtech with their existing IT systems. Even if these banks invest heavily on advanced technology, they do not tend to buy entirely new systems thus, such “fast and sexy solutions out there are almost useless” [57]. In determining whether to replace their old systems outright, banks will make informed decisions based on their existing business models and will only do so if the efficiencies generated by this technology clearly outweigh the risks [58]. Overall, this suggests that established financial institutions will be reluctant to scrap their existing systems to adopt Regtech even if their technology is not “readily adaptable, and often requires work in a proprietary language” [58]. This argument also holds true for regulators who may be sceptical towards disruptive technologies and refuse to abandon their current systems. Yet failure by regulators to integrate and adapt to the increasingly data-driven supervisory process may undermine the many years of effort to effectively implement and monitor regulatory compliance [10]. As a result, this prevents Regtech from becoming the catalyst of financial industry transformation given that the cumbersome integration and adaptation process may deter large financial institutions and regulators from actually adopting it.

More generally, proper Regtech integration necessarily entails integration in terms of legal language. An important question, therefore, is how to ensure complete Regtech integration with an ever-evolving legal and economic environment. How can the law be integrated with numbers? Given that regulations are constantly changing and becoming more complex, Regtech specialists must ensure that these rules are coded using legal language rather than programming code [59]. This means that the solution must be sufficiently ‘smart’ to develop a legal understanding, otherwise solutions “will quickly become obsolete” [59]. The current limitations of AI inevitably mean that Regtech solutions cannot understand and enforce natural language, but are rather confined to executing code. Current solutions thus require regular manual update to adapt to the dynamic regulatory framework. The lack of legal understanding thus limits Regtech solutions to being reactive rather than proactive.

It is important nevertheless to note that currently, the FCA’s Digital Regulatory Reporting envisions regulations to be issued both as natural language and as code [60]. This is certainly advantageous because “converting regulatory text to a machine-readable format using natural language processing leads to greater consistency and improved compliance” [13]. More specifically, provided this vision materialises, it could be argued that the issue of Regtech adaptability and proper integration will be solved. This is because machine-readable regulations will bridge “the gap between regulatory intent and interpretation” and “help supervisory agencies to efficiently assess the impact of regulatory changes, consult on regulatory reforms, and reduce regulatory complexity” [13]. This innovation is one example where Suptech and Regtech coexist, interact or even overlap to horizontally enhance oversight and regulatory compliance by regulators and financial institutions respectively. Consequently, if FCA’s vision becomes a reality, it is possible to expect Regtech to make a significant leap forward towards proper implementation and integration.

### Lack of international regulatory harmonisation and domestic implementation

A critical restraint to Regtech development is presented by the lack of international harmonisation. The effective development and widespread adoption of Regtech requires greater harmonisation of financial markets and regulations as well as international coordination. The harmonisation of financial markets and regulations carries a long history and has been at the regulatory epicentre due to the increased mobility of Fintech start-ups [8]. The primal reason for the existence of several international standard-setting bodies^5^ is to ensure regulatory harmonisation and international coordination. However, fintech does not fall cleanly under any specific standard-setting body. Fintech rather falls under all of them to a lesser extent, yet none are solely responsible for it. Currently, the laws and procedures applicable to Fintech solutions are ambiguous and inconsistent [50]. Yet to deliver a “streamlined process that consolidates multi-jurisdictional regulation demands in a single platform”, Regtech requires the international regulatory framework to be consistent [32]. Arner et al. suggested that the solution is to balance the views of technology specialists, financial institutions and regulators to develop an approach which is proportionate to their respective obligations [50]. However, this exercise necessitates an understanding of the “raison d’etre of regulators and the reasons behind the rules they enforce and to provide education for start-ups on their regulatory obligations” [50]. Evidently, this solution is not easy. It requires substantial international coordination and a systematic effort by market actors for uniformity.

Although there have been some efforts for an agreed internationally harmonised standard, these nevertheless require implementation in domestic legal systems of individual jurisdictions [6]. Prudential regulation requirements mandate that financial incumbents must produce the required data within a specific framework and timeframe specified by regulators [6]. This leads to a harmonised standard albeit with varying details on implementation across different jurisdictions [6]. Accordingly, even if regulators coordinate to tackle AML and international bodies comply with the minimum standards, the particulars of implementing these recommendations vary among jurisdictions, thereby undermining the efficiency of international coordination [61]. This is a critical issue for Regtech development as it gives rise to major compliance issues. For example, if individual jurisdictions have inconsistent regulatory requirements, this inevitably means that financial service providers operating in multiple markets have to utilise technological solutions that take into account both general global regulatory requirements as well as their individual requirements [6]. Yet Regtech solutions are currently limited to monitoring and automating compliance with particular regulations. If there are conflicts between regulations or varying details, financial institutions cannot be fully compliant solely by relying on Regtech. Consequently, until there is an “automated and proportionate regime … built on shared fundamentals of financial regulation” [61] Regtech is not able to transform the financial services industry as the regulatory framework itself is not consistent. At the same time however, it is important to note that even if the regulatory regimes are varying the basic information required by regulators would be mostly identical hence the modalities of presenting information can be designed in the Regtech.

### Possibility of a ‘black-box’ approach

Arguably, the most important limitation of Regtech is the possibility of discriminatory biases in the Regtech solution code. Micheler accurately noted that “biases present in the existing data set perhaps resulting from manual inspection regimes can easily be converted into automated biases” [62]. This produces the possibility to circumvent fundamental legal and constitutional principles. Given that machine learning is underpinned by ‘black-box’ decision procedures, Regtech specialists may not be able to determine whether a given solution operates on a bias or the direction that the bias follows [62]. Therefore, if the bias is not apparent, a Regtech solution could be operating on that bias without detection. One possible repercussion of Regtech bias could be a flood of unsubstantiated suspicious activity reports against the wrong individuals or firms. This risk is amplified in the case of Deep Learning Algorithms. Such algorithms are able to override their programming rules, taking decisions which are incomprehensible meaning that regulatory powers would effectively pass on to machines that feed themselves and define their own rules [63]. This results in an impossibility to determine the rules applied by the software in advance “with a consequent paradoxical violation of the principle of legal certainty which was precisely the aim pursued” by Regtech specialists [63].

The risk of circumventing legal and constitutional principles is also present in circumstances where financial institution employees identify and uncover the range of transactions flagged by Regtech solutions and maliciously use that information to circumvent regulatory detection [10]. Although this does not involve bias per se, it nevertheless demonstrates the scope for code exploitation. Overall, the inherent uncertainty and unpredictability of Regtech means that “we cannot simply rely on AI and machine learning as a panacea. We should not just accept the ‘black-box’ but ensure we look through the decision making and test this thoroughly” [64]. This implies that Regtech may not become a catalyst in the financial services industry transformation. In fear of these risks materialising, management will not simply delegate absolute compliance responsibility to Regtech solutions. Therefore, the human element cannot be eliminated ex post but rather, most responsibility will be retained by the management in an effort to minimise the effects of a ‘black-box’ Regtech solution [65]. This suggests that Regtech remains a regulatory tool which merely empowers compliance officers to move away from lengthy and repetitive administrative tasks to be able to concentrate on sophisticated tasks which require greater human analysis and judgment [64]. On the other hand, it is possible to argue that while it is true that the ‘black-box’ can be evolving, the reporting financial institution can take undue advantage of the gaps. Regulators deploying ‘black-box’ can consider its output as suggestive rather than decisive in order to mitigate the risks associated with the ‘black-box’ approach.

## The future of regtech: Mere facilitator or catalyst?

The COVID-19 crisis has already brought about catastrophic consequences across different sectors of the economy and Regtech should by no means count as an exception. Struggling businesses are not expected to invest in developing Regtech in-house or adopting existing Regtech solutions but rather use their depleting resources to survive. One of the vital lessons learned by the pandemic, however, is the importance of digitisation and automation for businesses to adapt in constantly changing environments. Between working from home, changing offices or even relocating, it is reasonable to anticipate a drive towards ‘pandemic-proofing’ by businesses. The physical constraints posed by the pandemic should encourage businesses to implement compliance automation and reporting technologies to adapt to the ‘new normal’. At the same time, the inefficiencies generated by the current regulatory landscape exert pressure on the market to utilise Regtech as the new regulatory norm. Regulatory burdens and budget limitations are driving financial incumbents and regulators towards replacement of human decision-making activities with automated solutions [10].

Businesses have always strived to increase efficiency within their reporting and compliance processes. It is therefore reasonable to wonder why they are not striving to make the processes themselves more efficient rather than relying on expensive technology. The processes could become more efficient through improved division of labour, extensive education and training as well as organised communication and collaboration with supervisory agencies. Although this would certainly achieve noticeable improvements, the reality is that the global drive towards automation is not without reason. If conducted prudently and correctly, compliance automation will always provide more efficient results as opposed to manual compliance which is limited by its nature due to its reliance on human input and output.

ESMA argued that with appropriate implementation and safeguards, Regtech is able to enhance compliance with regulatory obligations in a cost-effective manner whilst also assisting regulators monitor and analyse large and complex datasets [10]. However, this would also generate further legal and operational risks as regulators handle even-larger amounts of sensitive datasets [63]. It is thus impossible to predict how Regtech may in fact interact with the financial system. Indeed, “it may allow us to better manage risk leading to more stable financial institutions or it may turn out to steer us in the wrong direction” [62].

Although one cannot predict with certainty what the future holds for Regtech, it is nevertheless possible to discern two potential pathways. A likely possibility could see Regtech confined to a limited tool which merely assists financial incumbents to comply with certain regulatory requirements and regulators to handle large datasets. This possibility portrays Regtech as a subordinate tool which requires human oversight and monitoring because of the infinite ways it may backfire. An equally likely possibility could see Regtech becoming the catalyst in transforming compliance with regulatory requirements and analysis of big data by regulators. However, such possibility is contingent upon addressing some of the limitations explored in Sect. 4. This Section examines several recommendations to determine whether they adequately address some concerns so as to entrench Regtech as the driving force behind the financial industry transformation.

### Harmonisation and international coordination

The Expert Group on Regulatory Obstacles to Financial Innovation (‘ROFIEG’) has drawn up a report for the European Commission which comprises of thirty recommendations on regulation, innovation and finance. The relevant proposals on Regtech are Recommendations 9 and 10. ROFIEG acknowledges the efficiencies generated by Regtech and AI technologies. Specifically, they advocate that Regtech offers “greater accuracy and efficiency in reporting and data analysis and in turn, more timely risk analysis and mitigation” [2]. Secondly, they submit that Regtech achieves lower costs for financial institutions and faster regulatory reporting and data processing for firms and regulators [2]. Yet ROFIEG recognised that the current EU framework does not directly address Regtech resulting in an “ad-hoc and uncoordinated” development and implementation [2]. This lack of harmonisation has already been identified as a major impediment to Regtech development. Indeed, industry specialists interviewed for a particular study stressed the importance of semantic convergence and blamed the lack of shared standards and interoperability as the main impediment to industry expansion [66]. This view is sensible since a financial institution operating across a multitude of jurisdictions will be unable to provide the same Regtech solution for all subsidiaries because of “different supervisory expectations and technological capacities” [2]. As a result, Recommendation 9 advises the development and implementation of a comprehensive agenda by the EU in an effort to establish advanced Regtech capabilities “in coordination with relevant authorities in and beyond the EU and international standard setters” [2]. This would certainly constitute a positive step towards a more harmonised regulatory framework underpinned by advanced technology and greater international coordination. Nevertheless, a comprehensive regulatory agenda is not by itself sufficient to achieve substantial harmonisation. It will also require the support of international bodies, regulators and financial service providers to coordinate their perceptions, expectations and standards to achieve any meaningful reform to regulatory compliance.

Several other international organisations and academics have strongly advocated in favour of establishing Regtech as a harmonisation tool developed on shared fundamental principles of financial regulation. To begin with establishing Regtech in shared fundamental principles of regulation is right approach. With the high degree of integration of markets between differently regulated players, Regtech will soon have to be multi-regulator reporting platform. For instance, the Institute of International Finance (‘IIF’) produced a report on deploying Regtech against financial crime. IIF advocated that effective and efficient Regtech development for both AML and KYC requires closing gaps in the international AML framework, thereby providing a more harmonised and standardised international regulatory regime [11]. This generates numerous efficiencies such as improving communication between regulatory authorities and financial incumbents in different jurisdictions as well as achieving effective information sharing within financial groups and relevant authorities [11]. Likewise, adopting a common international approach would also contribute towards enhanced Fintech regulation. Arner et al. believed that this would “maximise market opportunity while at the same time setting best practices for managing risks to financial stability and consumer protection, similar to those that have been applied in the context of payment systems and other forms of regulation by international standard setters” [50]. Nevertheless, they advocated that the time for this approach is not yet ‘ripe’ because more “experimentation and innovation is needed in regulatory approaches and in Regtech” [50]. Although this may have been true in 2015 when the research paper was published, five years later one can reasonably submit that the time has come. Regtech industry has grew immensely in these past five years, regulators have increased awareness and innovation through sandboxes and financial service providers are turning en masse to automated regulatory solutions. In conjunction with the comprehensive EU agenda, a common international approach is vital in unlocking Regtech’s catalytic potential and in turn, transforming the ways in which market actors understand and comply with regulatory requirements.

A common international approach to Regtech also compliments the recent EU drive to create a new AML body to cover the entire internal market. Although the ECB has supervisory powers on prudential matters in the Eurozone, it is not equipped with financial crime controls [67]. The ECB suggested that this body would address supervisory fragmentation through a regulatory rulebook, coordinate its implementation and ensure strict and harmonised AML supervisory practices in the EU [68]. This body would also provide “timely assessments on possible irregularities” by proactively identifying and assessing financial crime risks for regulators [68]. Subsequently, it could also work together with Regtech specialists to educate regulators and financial incumbents on the merits of these solutions. Through addressing supervisory fragmentation and educating market actors on regulatory technologies, this body could instigate wider and uniform Regtech adoption.

### Standardisation

The implementation of ROFIEG’s Recommendation 10 is the final piece of the regulatory puzzle in unlocking Regtech’s potential to transform the financial services industry. ROFIEG urged the European Commission to promote the standardisation of legal terminology and the digital standards-based common classification of actors, services, products and processes in the financial industry [2]. This recommendation is premised on the view that a common language is necessary to facilitate digital communication among financial incumbents and regulators [2]. ROFIEG submitted that this approach is a “*conditio sine qua non* for interoperability between digital services and products, and to make regulation and supervision more efficient and effective” [2]. Without standardisation, the meaning of products, concepts and terms is determined individually by each financial institution thereby generating inconsistencies and thwarting efficiency, innovation and easy market access [2]. Therefore, Regtech must be underpinned by a common language and a common rulebook because otherwise, these solutions would “do little to solve fundamental problems … simply digitising the status quo” [2]. At the same time, however, the European Commission must be vigilant in promoting standardisation. If the European Commission goes beyond standardising terminology to standardise Regtech solutions, it may inhibit innovation incentives. If the same solution is provided across a particular sector, competitors will not be incentivised to innovate and create better alternative solutions [31]. For this reason, it would be beneficial if developing standardisation of Regtech components can be done through a body of a variety of regulators’ representatives. Furthermore, a decision in favour of software standardisation may bring about systemic risk. If a sub-optimal software is declared as the standard and deployed across a particular market, the consequences on the industry would be fatal [31]. This can be contrasted with the mild consequences of a flawed system relied upon by a single financial institution [31]. Thus, although the European Commission must promote standardisation of legal terminology and the digital standards-based common classification of actors, it should also be cautious to avoid potential backfires to Regtech’s potential.

### Embedded supervision

A distinct species of innovative technology has been articulated by Auer which relies on DLT to build regulation into blockchain finance. In essence, Auer argues that supervisory technology should evolve to generate new ways of supervising the ever-expanding DLT-based markets [69]. Auer manifested the concept of ‘embedded supervision’ which “comprises a regulatory framework that provides for compliance to be automatically monitored by reading the market’s ledger” and therefore, “reduces the need for firms to actively collect, verify and deliver data” [69]. This paper takes the view that the concept of ‘embedded supervision’ will impact the ways in which Regtech advances in the future. DLT-based markets allow for the “decentralised trading of asset-backed tokens, as well as decentralised financial engineering based on these tokens via self-executing (‘smart’) contracts” therefore enhancing the development of financial infrastructures through new forms of transparency and data credibility [69]. The distinguishing feature of DLT-based markets is that DLT “builds such credibility with a decentralised data structure based on economic consensus” effectively, replacing the need of market participants to trust third parties with the ability to trust the technology itself [69]. On this basis, Auer predicts that compliance monitoring by supervisory authorities would become automated and based on the reliance of this trust-creating mechanism of DLT-based markets.^6^

Henceforth, how will ‘embedded supervision’ affect Regtech development? Auer submits that “embedded supervision could ease the conflict between data availability, the cost of data collection and verification, and privacy” [69]. The implementation of ‘embedded supervision’ inevitably means that supervisory agencies would be able to obtain the data they need and at the same time reduce the costs of compliance for financial institutions. Overall, one may reasonably expect DLT-based markets to thrive in following years as market participants constantly explore and cultivate ways to exploit DLT. The outcome of Regtech’s interaction with ‘embedded supervision’ is limited to pure speculation. Currently, the most realistic possibility would see Regtech becoming overshadowed by ‘embedded supervision’. This is mainly because there would be no use for automating data gathering and reporting on DLT-based markets given that compliance would be automatically monitored by the market’s ledger thus, financial incumbents would not actively have to collect and deliver data. Consequently, this may mark the downfall of Regtech unless the technology finds a way to remain relevant and useful in DLT-based markets.

### Concluding remarks

Regulators have realised that a retrospective approach focused on the risks behind the previous crisis is not the way forward and therefore, now seek to enhance future market development and financial stability through technology [50]. In the same way that Fintech has reformed the operation of financial service providers, Regtech and Suptech are capable of transforming how financial institutions and regulators respectively comply with and oversee regulatory requirements [10]. The Regtech phenomenon is more than a mere buzzword. The efficiencies it generates both for the financial industry and regulators are immeasurable. This paper has nevertheless demonstrated how the risks and limitations associated with Regtech’s development, integration and operation have slowed down the envisaged widespread adoption. The adoption of Regtech may also be hindered in emerging DLT-based markets where ‘embedded supervision’ may eclipse the necessity for automating reporting and compliance technologies. Therefore, the emergence of DLT-based markets and the aftermath of the COVID-19 crisis, render the future of Regtech uncertain. Nonetheless, it is prudent to anticipate that the global push towards automation will play a vital role in shaping Regtech’s future. This together with a careful implementation of the aforementioned recommendations should enable international bodies, regulators and financial institutions to utilise Regtech to steer the regulatory spaceship in the right direction.

## Footnotes

1 For example, P2P Lending and Crowdfunding.

2 In 2017–2018, there was a 40% increase in the number of suspicious transaction reports compared to previous five years.

3 For example, greater automation in trading strategies.

4 Automation is the creation and application of technologies to produce and deliver goods and services with minimal human intervention. The implementation of automation technologies, techniques and processes improve the efficiency, reliability, and/or speed of many tasks that were previously performed by humans [53].

5 For example, Basel Committee on Banking Supervision (BCBS), International Association of Insurance Supervisors (IAIS) and International Organization of Securities Commissions (IOSCO).

6 For instance, compliance with the Basel III capital standards would be automatically verified in scenarios where financial institutions hold asset-backed tokens.
